# Determinants of Pulmonary Tuberculosis among Inmates at Mangaung Maximum Correctional Facility in Bloemfontein, South Africa

**DOI:** 10.1155/2015/752709

**Published:** 2015-03-17

**Authors:** Peter Nyasulu, Serame Mogoere, Teye Umanah, Geoffrey Setswe

**Affiliations:** ^1^School of Health Sciences, Monash University, 144 Peter Road, Ruimsig, Johannesburg 1794, South Africa; ^2^School of Public Health, Faculty of Health Sciences, University of the Witwatersrand, 7 York Road, Park Town, Johannesburg 2193, South Africa; ^3^HIV/AIDS, STI and TB (HAST) Research Program, Human Sciences Research Council, 134 Pretorius Street, Pretoria 002, South Africa; ^4^Department of Health Studies, University of South Africa (UNISA), 1 Preller Street, Muckleneuk, Pretoria 0002, South Africa

## Abstract

*Introduction*. Correctional facilities house large number of inmates who are at high risk of developing tuberculosis (TB); however factors associated with TB among inmates at Mangaung Correctional Centre have not been studied. *Study Population and Methods*. We undertook a case control study and reviewed a total of 1140 medical records of inmates treated for TB between 2009 and 2010. Cases were selected randomly from the medical records of inmates who were treated. Data collected were analysed using STATA version 12.0 and determinants of TB were evaluated using multiple logistic regression analyses. Factors with *P* < 0.05 were considered significant. *Results*. Prevalence of TB was 8.8% and 52% of inmates with TB were aged 31–40 years; 58% of the TB cases were HIV positive and 34% of them had CD4 cell count 350 cells/mm^3^. Factors associated with TB among inmates were HIV coinfection (OR: 4.2; 95% CI: 2.64–7.00); previous history of TB disease (OR: 3.58; 95% CI: 2.25–5.70); and smoking (OR: 2.1; 95% CI: 1.16–3.81). *Conclusion*. Interventions to improve TB detection such as regular screening of inmates with such factors need to be reinforced to control transmission of TB among inmates and the community.

## 1. Introduction

Prison systems worldwide have been known as focal points for the concentration and dissemination of tuberculosis (TB) amongst inmates. Prisons are increasingly becoming breeding grounds from which infection is transmitted to the general population and accounts for up to 25% of a country's TB burden [1]. An estimated 360,000 inmates move through the South African correctional centre system annually; this movement worsens the spread of communicable diseases especially TB [2]. The spread of TB between prisoners, staff, and visitors and the emergence of drug-resistant TB in prisons now threatens the control efforts of national tuberculosis programmes in sub-Saharan Africa (SSA).

TB among prisoners in this region is not well documented despite its high endemicity. Accurate data of TB in prisons in SSA countries are not readily available since surveillance and data reporting mechanisms are poor or nonexistent. While there have been many comprehensive literature reports of TB in prisons from USA and Europe, very little research has been done in developing countries including SSA on the risk factors associated with the incidence and spread of TB in the correctional facility settings [3].

Although precise information on prison conditions and the burden of the disease in prisons in Africa remains scarce, available data suggest that many of these facilities have outdated infrastructure and are overpopulated. The situation is even more disturbing given the difficult living conditions, extreme overcrowding, poor ventilation, poor sanitation and hygiene, poor nutrition, and substandard health care. These conditions contribute to the high prevalence and transmission of TB in prison settings [4]. According to WHO (2014), the prevalence of TB in prisons is up to 100 (range: 10–100) times higher than that of the civilian population, in both low- and high-TB burden countries [1]. Studies carried out in Ivory Coast, Malawi, Botswana, California, and Bangladesh state that prisons found TB prevalence of 10 to 35 times higher in prisoners than in the general population [4]. A 3- to 1000-fold increase in the prevalence of TB in prisons compared to the general population has also been documented in a recent systematic review [5].

A prospective study of TB in Cameroonian prisons showed an annual incidence of active TB to be 1700 cases per 100,000 person-years [6]. In a Ugandan study, the reported TB incidence was 955 per 100,000 person-years [7], which is nearly 20 times higher (505 per 100,000) than that of the general population reported in Columbia [8].

Several factors have been implicated as determinants of TB infection in prisons and these include younger age compared to older age groups [9, 10]; malnutrition (defined as body mass index (BMI) < 18.5 kg/m²) [5, 8, 11–13]; high risk behaviour such as substance abuse and injection drug usage [14–16]; and smoking [11, 12, 17]. Additionally, previously diagnosed/treated TB [8, 18, 19], exposure to TB patients, previous imprisonment [11], and longer duration of imprisonment [9, 11, 14, 20, 21] were associated with development of TB in prisons.

The architecture of prisons and the general living conditions in these facilities increase the risk of TB disease. Prison construction plans invariably focused on security as higher priority than adequate ventilation. Various studies showed that in situations where inmates with active TB live in poorly ventilated custodial settings the potential for rapid transmission of TB was high. The overcrowded conditions of most prisons and poor ventilated close quarters in which inmates are housed facilitate the transmission of TB. These factors may often lead to TB outbreaks in the prison systems [15, 20, 22]. Although overcrowding in prisons increases the risk of TB, it will not be discussed in this study as the inmates in this centre are housed according to set norms.

In SSA, the region most affected by Human Immunodeficiency Virus (HIV) infection, extraordinarily high rates of HIV have been documented in prison populations. HIV is a major predictor for tuberculosis, with HIV positive individuals being at an estimated 20 times higher risk of developing TB during their lifetime. The high prevalence of TB disease in prisons has also been associated with the HIV/AIDS epidemic [1]; and several studies in the developed and developing countries have demonstrated high prevalence of HIV in prison populations [9, 12, 23, 24] with HIV being a strong predictor of developing TB in prisons [5, 13, 14, 19]. Inmates with suppressed immunity are more likely to develop TB than those with normal immunity. Low CD4 cell counts due to HIV infection have been associated with the development of TB disease [22].

In view of these factors, it is important to determine the prevalence and risk factors for TB infection in prisons as outbreaks will not only affect the inmates, but also spread to both the prison personnel and medical team and invariably the general population at large. This study aimed to identify risk factors associated with TB among inmates in the Mangaung Correctional Centre.

## 2. Methodology

### 2.1. Study Design

The study used a retrospective case-control analytical design to review existing data from the medical records of inmates diagnosed and treated for TB compared to those with no history of TB for the period of the study.

### 2.2. Study Population

The study population was male inmates who presented at the clinics and were treated for any aliments including TB at Mangaung Correctional Centre between July 2009 and June 2010. Data were sourced from available medical records and TB registers.

### 2.3. Sampling

Sampling was designed to identify the TB cases and non-TB cases (controls). A total of 1140 medical records were retrieved and reviewed. One hundred (*n* = 100) of the inmates were identified as TB cases from TB registers and 1040 non-TB controls were randomly selected from patients who were treated at each of the housing unit clinics and the health care centre between July 2009 and June 2010. TB cases were all those treated for TB on the basis of any combination of history, exam, and smear characteristics. Based on this case definition, patients with intrathoracic, nonpulmonary TB (e.g., mediastinal or pleural TB) were not included. The controls were selected based on absence of history of chronic cough, previous or ongoing symptoms, and signs of TB. Systematic random sampling was applied for selecting the controls in the study.

### 2.4. Study Site

Mangaung Correctional Centre is a privately administered prison situated in Bloemfontein, the capital of the Free State province. It is run by a British based company Global Solutions Correctional Services in partnership with the South African Government Department of Correctional Services. This is a maximum security facility exclusively for male offenders with moderate to severe criminal charges, serving long term sentences. The centre is one of 11 maximum facilities distributed throughout South Africa. The prison consists of six housing units designed to accommodate 488 inmates per unit. The units are subdivided into cells with two or four inmates sharing a cell. At any given time the number of inmates housed in a unit may vary as some inmates will be admitted at the health care facility or sent to solitary confinement (isolation units) due to bad behaviour. There is a total population of 2928 male inmates from all nine provinces and from neighbouring countries like Lesotho, Zimbabwe, and Mozambique.

Health care services are provided by Faranani Life Health Solutions which employs doctor driven primary health care approach. Health care provision is mainly on site with a 54-bed inpatient health care facility and covers all acute, chronic, and emergency aspects of care to inmates. Out-patient primary health care clinic services led by professional nurses are provided to each housing unit on a daily basis, with doctor visits once a week.

The facility also offers initial TB screening to inmates at admission based on history and physical examination with sputum microscopy using Ziehl-Neelsen or fluorescent auramine staining. This was performed on sputum of patients presenting with chronic cough as a form of active case finding. During incarceration, passive case finding is applied to diagnose TB among inmates who present at the prison clinics. Diagnosed TB patients are normally admitted to the health care facility for the first two weeks of their TB treatment and then discharged to continue treatment at unit clinics. Treatment with antiretroviral drugs is also provided to those inmates with HIV coinfection. Health care provision is not limited to inmates; prison employees are also consulted on a need basis and during preemployment medical examinations and emergencies. There are over 400 employees who receive health care services on site. The full time radiographer and psychologist and part time consultants in surgery and orthopaedics, specialist physician, psychiatrist, and urologist are contracted to support the primary health care doctors and professional nurses.

### 2.5. Measurements

Appropriate variables were categorised into immune and nutritional factors, risky behaviour, and incarceration factors; the following variables were included:* immune and nutritional factors*, that is, Human Immunodeficiency Virus (HIV) coinfection, CD4 cell count (cells/mm^3^), and BMI (kg/m^2^) proxy for nutritional status;* risky behaviour*, that is, substance abuse (involving injection drug use, narcotics, other locally available drugs, and alcohol abuse); and* incarceration factors*, that is, length of stay in prison (years). Potential confounders included age in years, smoking status, and previous TB history.

### 2.6. Data Collection and Analysis

Data were abstracted from existing patients medical records and entered into a predesigned excel data capture sheet. Data were then cleaned assessing completeness and appropriate coding was done. Clean data were exported to STATA version 12.0 (Stata Corp, College Station, TX) for analysis. Data for age and length of stay in prison were categorised and presented as categorical variables. The relationships between risk factors and TB were evaluated using bivariate and univariate analyses. Categorical variables were compared using Pearson *χ* ^2^ test or Fisher's exact test where appropriate. A multiple logistic regression analysis was performed to determine significant risk factors for TB disease among inmates and odds ratios were used as measure of effect with corresponding 95% confidence interval.

### 2.7. Ethical Approval

The study was approved by the University of the Witwatersrand Committee for Research on Human Subjects (Medical) with clearance certificate number M10832. Other approval and support letters to conduct the research in prison were obtained from the prison authorities and the clinic management. To maintain confidentiality and anonymity, there was no contact with the study participants and no individual identifiers were used. Data and information obtained from the medical records were coded and only accessed by the researcher. The findings of the study were made available to the prison authorities and the clinic management so that appropriate interventions could be implemented to decrease TB among inmates.

## 3. Results

### 3.1. Distribution of Study Characteristics

A total of 1140 medical records of male inmates were reviewed, *n* = 100 inmates had active TB disease (TB cases) and *n* = 1040 inmates had no TB (non-TB cases), and all records reviewed were included in the analysis. This gave a TB prevalence of 8.8% (*n* = 100/1140) among the inmates studied. The majority (42.6%) of the inmates in this study population were aged between 31 and 40 years. The mean age was 35.7 years with a range of 22–67 years. The median length of stay in prison was 5 years with a range of 1–22 years.

Table 1 shows the age grouping between the TB cases and non-TB cases. Thirty-three percent (*n* = 33) of the TB cases were aged 21–30 years compared with 27.0% (*n* = 281) of non-TB cases. Over half, 52% (*n* = 52), of TB cases compared with 41.7% (*n* = 434) non-TB cases were found in the age group 31–40 years. More inmates, 22.5% (*n* = 234), had no TB in the older age group 41–50 years, compared with the 14% (*n* = 14) who had TB. The differences between the two groups were significant (Fisher's exact test *P* = 0.001).

The TB cases had more exposures of the immune and nutritional factors compared to the non-TB cases (Table 1). Forty-seven (47%) of the TB cases had low BMI of below 18.5 kg/m^2^ which is associated with malnutrition. 58% (*n* = 58) of TB cases were HIV positive compared to 18.5% (*n* = 192) non-TB cases, for those inmates with compromised immunity, and 44.6% (*n* = 463) of non-TB cases did not know their HIV status. 34% (*n* = 34) of TB cases had low CD4 cell count of below 350 cells/mm^3^ compared to 8.1% (*n* = 84) non-TB cases. The difference in the two groups was statistically significant *P* < 0.05. Smoking in the two groups was high with 49.2% (*n* = 512) of non-TB cases and 45% (*n* = 45) TB cases being smokers. But 19% (*n* = 19) of TB cases were ex-smokers compared to 10.2% (*n* = 106) non-TB cases (Table 1).

The risky behavioural factors among inmates that is, substance abuse was high in TB cases with 43% (*n* = 43) reporting using drugs compared with 29.7% (*n* = 309) non-TB cases, *P* = 0.018, as shown in Table 1. The increased length of stay in prison between the two groups was comparable (Table 1). The difference between the groups was not statistically significant, Fisher's exact *P* = 0.218.

### 3.2. Risk Factors Associated with TB among Inmates

In Table 2, the younger age group 21–30 years had increased odds of contracting TB. The odds ratio was borderline and not significant (OR: 1.02; 95% CI: 0.64–1.62) for the age group 31–40 years. The older age group 41–50 years had lower odds of contracting TB (OR: 0.51; 95% CI: 0.27–0.97). The inmates with BMI above 18.5 kg/m^2^ had less odds (OR: 0.21; 95% CI: 0.14–0.32) of developing TB than those with BMI below 18.5 kg/m^2^. Being HIV positive increases the odds of developing TB by four times (OR: 4.2; 95% CI: 2.64–7.00) more than the HIV negative status. Lower CD4 cell count ≤350 cells/mm^3^ was associated with TB but did not necessarily increase the odds of contracting TB (OR: 1.83; 95% CI: 0.98–3.39). The odds of TB were borderline (OR: 1.03; 95% CI: 0.65–1.63) for smokers and twice as high (OR: 2.1; 95% CI: 1.16–3.81) for ex-smokers as compared with nonsmokers. The result for smokers was borderline because of the confounding nature of smoking as an exposure factor. Previous history of TB disease triples the odds of contracting TB (OR: 3.58; 95% CI: 2.25–5.70). Substance abuse also increased the odds of TB by 1.87 times (OR: 1.87; 95% CI: 1.21–2.92), as shown in Table 2.

## 4. Discussion

This study showed a high prevalence of TB disease 8.8% (8772 per 100 000) among inmates in the Mangaung Correctional Centre. This prevalence was nine times higher than the total TB prevalence of the general population which was 948 per 100 000 as reported in 2009. High prevalence observed in this prison is in line with studies carried out in Cameroon, Malawi, Tanzania, Botswana, and East Ethiopia, where TB prevalence in prisoners was 7 to 35 times higher than in the general population [9, 10, 18, 23, 25] and between 3 and 1000 times higher as reported in the recent systematic review [5]. The following factors were identified as significant contributors of TB disease among inmates: age 21–30 years, HIV coinfection, BMI ≤18.5 kg/m^2^, CD4 cell count ≤350 cells/mm^3^, smoking (ex-smoking), previous TB history, and substance abuse. There was a significant association between age and the risk of contracting TB. Inmates aged 21–30 years were at higher risk of developing TB than those of older age. Thirty-three (33%) inmates aged between 21 and 30 years and 52% (*n* = 52) of age group 31–40 years had TB in this study. This finding is similar to those of other studies in east Ethiopian prisons where TB prevalence was higher among prisoners aged 15–44 years (13.4%) compared with older age groups (4.8%). TB was also found to be more prevalent among male inmates with the average age of 30 years, at Zomba Central prison in Malawi [9, 10].

HIV infection is a significant risk factor for TB disease. A four times increased odds (OR: 4.2; 95% CI: 2.64–7.00) of developing TB disease was found among inmates with HIV coinfection in this study. Of the TB cases studied 58% (*n* = 58) were HIV positive. It is well documented in previous studies conducted in other prisons worldwide that HIV coinfection increases the risk of TB among prisoners. It is also reported that HIV increases the likelihood of reactivation, reinfection, and progression of latent TB to active disease. A recent coinfection rate of 27.4% has been documented in Zambia [24]. In Tanzania, Bugando Medical Centre HIV coinfection of 25.9% (*n* = 433) was recorded among inmates with TB from Butima prison [23]. A high HIV seroprevalence of 73% (*n* = 45/62) was reported in a study in Malawi [9]. A study in Chicago CCDOC county jail revealed that TB was three times higher (OR: 3.07, *P* < 0.01) among HIV positive inmates [14]; and even higher odds of developing TB associated with HIV by self-report were noted in Tajikistan prison [19].

Low CD4 cell count ≤350 cells/mm^3^ were associated with 1.83 times higher odds of developing TB in this study. This observation is similar to that of California state prison where HIV infected inmates with CD4 cell counts <100 cells/mm^3^ were found to be at higher risk of developing TB disease. This confirms that inmates with suppressed immunity are more likely to develop active TB disease than those with normal immunity [22]. Malnutrition as defined by BMI of <18.5 kg/m2 was associated with the risk of developing TB. Forty-seven (47%) of TB cases in this study had low BMI of ≤18.5 kg/m^2^ compared with only 15.3% (*n* = 1040) of non-TB cases. It is well known that the association between malnutrition and TB disease is bidirectional, as malnutrition compromises host immunity and predisposes to TB inversely TB itself can cause malnutrition. The study finding that inmates with low BMI are more likely to develop TB compared with those who had normal BMI of >18.5 kg/m^2^ is similar to previously published literature [5, 8, 11–13]. In a prison in Bangladesh, the authors found that malnourished inmates were five times more likely to have TB. The likelihood of acquiring TB was 11 times more in inmates with severe malnutrition, BMI of <17 kg/m^2^ [11]. Malnutrition was also associated with TB disease in a penal camp of Bouaké, Ivory Coast, where 75% (*n* = 108) of TB infected inmates had malnutrition [12].

Thirty-one (31%) of TB cases studied, had a history of previous TB disease compared with 11.2% (*n* = 1040) non-TB cases. This study revealed that the odds of TB disease were three times higher (OR: 3.58; 95% CI: 2.25–5.70) for those inmates with history of previous TB. This result was also observed in a number of previous studies. A high prevalence of active TB was noted for patients with previous history of TB (prevalence ratio: 10.21; 95% CI: 6.27–16.63) in Tajikistan [19]. In Cameroon, previous TB disease was associated with high odds (OR: 4.06; 95% CI: 1.70–9.71) of developing recurrent TB [18]. Those inmates with history of TB disease had three times higher odds of developing the disease than those without TB history, as previously reported in Bangladesh and Columbia [8, 11].

Smoking is a well-known risk factor for a number of diseases. Smoking is associated with high risk of TB as reported in previous literature. Lower odds of developing TB among smokers (OR: 1.03; 95% CI: 0.65–1.63) compared with nonsmokers were found in this study. Ex-smokers were highly likely to develop TB disease (OR: 2.10; 95% CI: 1.16–3.81) and this was a statistically significant association. A study in Dhaka central jail, Bangladesh, also reported a strong association between smoking and TB with 85.7% of TB cases smoking at least five cigarettes per day [11]. In Ivory Coast, 52% (*n* = 108) of prisoners with TB disease were smokers [12]. A number of previous studies have reported smoking associated with increased risk of developing TB disease despite statistically insignificant results [17]. This could probably be due to the fact that smoking is a confounding factor in the development of TB disease.

Substance abuse was observed in 43% of TB cases in this study. The risk of TB was 1.87 times higher among substance abusers than nonabusers. This result is supported by a number of previous studies where drug use among inmates was associated with increased risk of TB disease. The risk of having TB among inmates studied in remand prisons in Saint Petersburg, Russia, was more than two times higher (OR: 2.6; 95% CI: 1.1–6.2) among those who used narcotic drugs [15]. In Chicago, in Cook County Department of Corrections (CCDOC) jail study 27.7% (*n* = 441) of the TB group were IV drug users as compared with 14.1% (*n* = 478) of non-TB group [14]. A review on HIV coinfected injection drug users revealed a 2–6 times higher risk of developing TB among these inmates compared with those not abusing injection drugs [16].

In this study the length of stay in prison was not significantly associated with contracting TB (Fisher's exact test = 0.218, *P* > 0.05), despite previous studies reporting either short or long stay in prison as risk factors for TB [11, 14, 20, 21]. Most studies reported that development of TB disease was significantly associated with the first six months of incarceration among prisoners. This could be because prisoners come from high risk communities with high prevalence of TB and may be already exposed to* Mycobacterium tuberculosis* infection prior to incarceration. Previous imprisonment and pretrial jail stay were also identified as risk factors for developing TB [11, 14, 21]. In general inmates stay longer in remand jails or police holding cells awaiting trial, due to the prolonged criminal justice process of court days and delayed sentencing. These factors were not considered in this study, mainly because of limitations of the data source.

The present study is the first to investigate the risk factors associated with TB among the inmate population in South Africa. The study identified the risk factors and their degree of association with TB disease while in prison at Mangaung Correctional Centre; however these factors may not be limited to this prison only; they could also be generalised to other prisons in South Africa. These findings would help to enhance screening and diagnosis of TB in prisons and timely institution of TB treatment to control transmission of TB infection in correctional facilities. Certainly the results shown in this study highlights the urgent need for policy changes targeting the prison environment so as to contain the spread of TB within and outside the prison boundaries by undiagnosed inmates who may have been discharged from prison and gone to their communities. Prison authorities in liaison with the National Department of Health needs to develop prison-specific interventions which are integrated into the TB control strategic framework of the National TB control Programme. This study forms a baseline platform from which more elaborate investigations need to be conducted to further characterise the risk factors for development and spread of TB in prisons.

In considering the findings of this study, the following limitations need to be highlighted:

 A retrospective case-control study design is limited by the data source, in this case the medical records. Information that can be retrieved from these records is mostly clinical; details about socio-demographic characteristics like race, level of education, employment, and marital status or incarceration factors like pretrial jail stay and previous imprisonment are not contained in medical records. This kind of information is only available in prison records which were inaccessible for this study. High population turnover in prison was also another limitation; inmates are continuously being transferred to other facilities or released on parole; this meant their medical records were not available for investigation. Medical records of deceased and released inmates are not kept at this prison but are handed over to the Department of Correctional Services which then becomes property of the state.

## 5. Conclusion

This study is the first to report the risk factors associated with contracting TB in this prison. The findings that inmates are at an increased risk of developing TB disease are in line with earlier studies conducted in prisons in other countries. The high prevalence of 8.8% observed in this prison confirms that TB disease among prisoners is a serious health problem that should no longer be ignored. Prisoners are the most vulnerable individuals yet the most marginalised population in our society.

Prisoners have the right to receive better healthcare. Their health status cannot be separated from their behaviour and living conditions. Therefore appropriate and systematic interventions need to be developed and implemented immediately to control the development and transmission of TB disease in this high risk environment and prevent the spread to the general population. Implementation of infection prevention protocols and procedures in prisons targeting various levels, that is, environmental and administrative as well as personal protection aspects, is needed. For effective TB control in prisons and the prevention of TB transmission to the general community, a multifaceted collaborative approach is required between healthcare systems both inside and outside prisons. Considering the high population turnover in prisons, it is important to ensure that inmates, who are released or transferred before completion of their treatment, are able to continue treatment.

Standard recommendations of infection control and prevention, isolation of patients, and protection of medical staff apply to all institutions. However these are not implemented in most prisons in sub-Saharan African countries [4]. A closer look at the risk factors associated with TB in this prison gives some indication of where targeted interventions need to be focused. The identified risk factors which are: age 21–50 years, HIV coinfection, low BMI of ≤18.5 kg/m^2^, low CD4 cell count ≤350 cells/mm^3^, previous history of TB disease, smoking (especially ex-smokers), and substance abuse, should help target the high risk groups among inmates that must be closely monitored for developing TB. These findings call for the promotion of timely HIV counselling and testing, with provision of supportive care for coinfected inmates as well as early access to antiretroviral therapy. Therefore periodic TB screening especially with the use of GeneXpert MTB/RIF test which allows for timely diagnosis as well as, treatment initiation, and treatment completion are critically important among inmates so as to effectively control the spread of TB disease in correctional facilities.

## Figures and Tables

**Table 1:** Comparison of characteristics of study participants.

Characteristics	TB cases *n* = 100 (%)	Non-TB cases *n* = 1040 (%)	*P* value
Age (years)			
21–30	33 (33.0)	281 (27.0)	0.001
31–40	52 (52.0)	434 (41.7)	
41–50	14 (14.0)	234 (22.5)	
≥51	1 (1.0)	91 (8.8)	
BMI			
Below 18.5	47 (47.0)	159 (15.3)	0.0001
Above 18.5	53 (52.0)	881 (84.7)	
HIV			
Negative	27 (27.0)	384 (36.9)	0.0001
Positive	58 (58.0)	192 (18.5)	
Unknown	15 (15.0)	463 (44.6)	
CD4 count			
>350	21 (21.0)	95 (9.1)	0.0001
≤350	34 (34.0)	84 (8.1)	
Unknown	45 (45.0)	861 (82.8)	
Substance abuse			
No	45 (45.0)	621 (59.7)	0.018
Yes	43 (43.0)	309 (29.7)	
Unknown	12 (12.0)	110 (10.6)	
Smoking			
Nonsmoker	36 (36.0)	422 (40.6)	0.026
Smoker	45 (45.0)	512 (49.2)	
Ex-smoker	19 (19.0)	106 (10.2)	
Previous TB			
No	69 (69.0)	924 (88.6)	0.0001
Yes	31 (31.0)	116 (11.2)	
Duration in prison (years)			
1–5	11 (11.0)	72 (6.2)	0.218
6–10	45 (45.0)	395 (37.9)	
11–15	26 (26.0)	312 (30.0)	
16–20	13 (13.0)	169 (16.3)	
>20	5 (5.0)	92 (8.9)	

**Table 2:** Risk factors and degree of association (odds ratios).

Risk factor	Odds ratio	95% CI
Age (years)		
21–30	1	
31–40	1.02	0.64–1.62
41–50	0.51	0.27–0.97
≥51	0.65	0.08–5.17
BMI		
Below 18.5	1	
Above 18.5	0.21	0.14–0.32
HIV		
Negative	1	
Positive	4.2	2.64–7.00
CD4 count		
>350	1	
≤350	1.83	0.98–3.39
Smoking		
Nonsmoker	1	
Smoker	1.03	0.65–1.63
Ex-smoker	2.10	1.16–3.81
Previous TB		
No	1	
Yes	3.58	2.25–5.70
Substance abuse		
No	1	
Yes	1.87	1.21–2.92
